# How Fast Are the Asian Countries Progressing Toward Green Economy? Implications for Public Health

**DOI:** 10.3389/fpubh.2021.753338

**Published:** 2022-02-07

**Authors:** Ming Shao, Hui Jin, Fu-Sheng Tsai, Mihajlo Jakovljevic

**Affiliations:** ^1^School of Management, Shanghai University of Engineering Science, Shanghai, China; ^2^School of Economics and Management, Zhejiang Sci-Tech University, Hangzhou, China; ^3^North China University of Water Resources and Electric Power, Zhengzhou, China; ^4^Department of Business Administration, Cheng Shiu University, Kaohsiung, Taiwan; ^5^Center for Environmental Toxin and Emerging-Contaminant Research, Cheng Shiu University, Kaohsiung, Taiwan; ^6^Super Micro Mass Research and Technology Center, Cheng Shiu University, Kaohsiung, Taiwan; ^7^Department Global Health Economics and Policy, University of Kragujevac, Kragujevac, Serbia; ^8^Institute of Comparative Economic Studies, Hosei University Faculty of Economics, Tokyo, Japan

**Keywords:** green economy, measuring progress, entropy method, indicators, environmental assessment, Asia

## Abstract

Monitoring progress toward green economy has been a key policy focus globally. The purpose of our study is to assess Asian countries' green development performance and also the progress toward green economy overtime. To achieve this goal, we propose a green development index (GDI) to assess the level and ranking of green development for Asian countries, and then we measure the progress toward green economy by the method based on the compound annual growth rate (CAGR). The result shows that the northeast Asian countries together with Singapore and Israel are leaders in green development performance across Asia, but the most progress toward green economy has been achieved by some medium green development level countries, like China. Countries with the fastest movement away from green economy are some laggard countries with poor green development performance, such as Syria and Yemen. More generally, the leading countries have reached a high green development level, and the medium ones move fast toward green economy, whereas some laggards get worse. We also discuss the implications for public health in environmental protection, green consumption, and green production.

## Introduction

Monitoring progress toward green economy or green development has been the focus of both researchers and international organizations (1–3). Many international organizations and statistical institutions are continuously focusing on the implementation of green development or green economy policies, like the UN Statistical Commission, Eurostat (4, 5). Some researchers also attempt to set SDG indices for green development assessment (6, 7). In addition, there are many composite indices built to measure the performance and ranking of green development, such as the human green development index (8).

In existing green economy studies, the focus is often the global or European countries' assessment and ranking at a certain time point or period. Throughout these assessments and rankings, European and other OECD countries tend to come out on the top, whereas African countries on the bottom (8, 9). Within the Europe, the best green development performance is always found in the Scandinavian countries (10, 11). However, although these studies measure the green development level at a certain time point or period, they do not propose a method to indicate whether countries have moved toward or away from green economy over time and how fast they have moved, which is also very important (12).

Recently, a growing list of studies have pointed the importance of monitoring progress toward green economy over time and proposed a new method to measure it (3, 5, 13). This method, based on the compound annual growth rate (CAGR), could be used to assess development over time also in the absence of quantified policy targets (12). This CAGR method is adopted to study the EU members' progress toward SDGs, which has resulted that strong movement toward SDGs is found in those southern and eastern European countries with relatively low sustainable development level (Hametner and Kostetckaia).

However, Asia, as the largest continent with more than 4 billion people, has not received enough attention in studies of green economy measurement and progress. Some studies have measured the green development level of some Asian countries, like 12 developing Asian countries (14) and 6 Southeast Asian countries (15). Koirala and Pradhan (16) studied the determinants of green development in 12 Asian countries for the 1990–2014 period. But there are still many issues concerning the green development of Asian countries that need attention: (1) What is the green development performance and rankings of Asian countries? (2) Which countries are moving forward or away from green economy? (3) And how fast? (4) Is there a relationship between a country's green development level and the rate of progress toward green economy? (5) What's the implications for public health? These research questions are attempted to be answered in this paper.

The results of this paper are relevant for both researchers and policy-makers. First, this paper supports the viewpoint of Hametner and Kostetckaia (3), that is, it is not sufficient to calculate composite development indices and rankings at a time point or period, and it is necessary to monitor progress toward green economy over time. Second, our research will help to strengthen the understanding of green economy and green development. Many studies have paid attention to the environmental dimension while ignoring the essence of green development, namely coordinated development in economic, environmental, and social dimensions. Last, the result shows that the laggard countries in Asia move the fastest away from green economy, which implies that it is important to help these countries improve green development level.

The rest of the paper is organized into five sections. Section 2 is literature review. Section 3 describes the methods for measuring the green development performance and the progress toward green economy, as well as the data source. Section 4 is result, namely the green development ranking and progress score. Section 5 is a comparison between GDI and other similar composite indices. Section 6 concludes and derives some implications for public health.

## Literature Review

### Green Economy and Green Development

Since the “green economy” has been put forward by Pearce et al. (17), it has gradually become the focus of governments and researchers. As Pearce et al. (17) have pointed, green economy is an economic development mode that the natural environment and human beings can bear, which does not lead to ecological crisis and social division due to the pursuit of economic growth, and which does not lead to unsustainable economic development due to the depletion of natural resources. Also Reardon (18) further defines “green economy” as “the maximization of human happiness under the constraints of resources and ecology.”

Therefore, green economy is a kind of “green development” mode that coordinates economic and environmental development (8). The core of green development is “give consideration to both green and development,” and the goal is to achieve the harmony of green and development (19). On one hand, if were to only protect the environment and make the economy stagnation, this would not represent a green development mode. On the other hand, if were to promote economic growth at the cost of environment, that would also not be a green development (9). Green economy and green development are proposed based on the contradiction between economic growth and resources, environment and ecology, but not only does it mean to deny economic growth, but it also helps to seek a new way of economic growth (8). Stable economic growth, sustainability of resources and environment are very important for both developed and developing countries (20).

### Indices for Green Economy Assessment

A growing list of indices have been proposed for green economy or green development assessment (21). The indices in early times put more attention on environmental sustainability. For example, Hall and Kerr (22) proposed the Green Index to monitor the national environmental health in USA. Recently, scholars begin to pay attention to the coordinated development of environment and economy. Kumar and Kumar (23) suggested that developing countries can achieve low-carbon development through low pollution and high efficiency production technology. Halle (24) argued that accountability mechanism played an important role in the green development. Li et al. (8) built the human green development index by adding indicators of resources and environment to the Human Development Index. In addition, some international organizations also built green economy assessment indices, such as the WAVES (25), the green economy index (26), etc.

## Methods and Data

### Methods for Green Economy Measure: Green Development Index

According to the concept of green economy and the existing studies, this paper established the Green development index (GDI) with two dimensions, namely economic-social dimension, and resource–environmental dimension.

#### The Framework of the Green Development Index

According to the definition of green economy, the government should set green development as a comprehensive goal including economic–social and resource–environmental dimensions. Therefore, a composite index for green development assessment should include these dimensions (27). Jin et al. (9) suggested that a green development index should contain these factors, namely “economic growth,” “income level,” “education,” “health,” and “economic structure” in economic–social dimension, “climate,” “air quality,” “forest,” “arable land,” and “energy” in resource–environmental dimension. They believe that these are the basic indicators to coordinate the common development of human society and natural environment, and also the basic goal of human pursuit of green development.

The GDI is an attempt to be a concise, acceptable, and complete index, and so the five principles below should be followed when selecting the corresponding indicator for each factor: (1) indicators should be representative, and preferably mature and stable ones in existing studies; (2) the quantity of indicators should not be too many, making the composite index concise and acceptable (8); (3) indicators should be continuous and comparable over time (28, 29); (4) indicators must be quantifiable and have strong operability; (5) availability and reliability of the source of data (30). According to the principles above, we select one indicator for each factor so as to build the framework of GDI (see Table 1), while the selection process of indicators is in the next section.

**Table 1:** The structure of green development index.

**Index**	**Dimension**	**Factor**	**Indicator**	**Premise**
Green development index (GDI)	Economic- social dimension	Economic growth	Real GDP growth	+
		Education	Expected years of schooling	+
		Health	Life expectancy index	+
		Income level	Income index	+
		Economic structure	Employment in services (% of total employment)	+
	Resource- environmental	Climate	CO_2_ emissions per capita	–
	dimension	Air quality	PM2.5	–
		Forest	Forest area (% of total land area)	+
		Arable land	Arable land per person	+
		Energy	Renewable energy consumption (% of total final energy consumption)	+

*(1) The weight of each indicator is given by the Entropy Method described below. (2) The descriptions and data source of the 10 indicators can be found in Appendix Table A1*.

#### The Selection Process of Each Indicator

The representativeness and typicality of the selected indicators (variables) are related to the measurement and practical value of the GDI. Thus, it was very important to choose one indicator in each of the 10 areas related to green development. According to the criteria for choosing indicators and referring to the advanced practices of well-known indices, we formulated meticulous operation steps for indicator selection. Taking the selection process of the “Education” indicator as an example, the details are as follows.

(1) Searching relative indicators

There are more than 20 indicators for the factor of “education,” such as “government expenditure on education,” “government expenditure per student,” “gross intake ratio in first grade of primary education,” “literacy rate (adult),” “progression to secondary school,” “school enrolment, secondary,” “primary school enrolment,” “trained teachers in primary education,” “primary completion rate,” “mean years of schooling,” and so on. We studied and compared these indicators and chose the most representative and suitable indicator in each field based on the selection criteria and existing well-known indices.

(2) Comparing all indicators

According to the indicator selection criteria above, we compared all indicators and investigated their representativeness, comparability, continuity, and availability. For example, “government expenditure on education” can represent government spending and emphasis on education, but cannot effectively measure current education quality and future education development. The data for “trained teachers in primary education” are not available in more than 120 countries. Fortunately, these indicators for education are all continuous and comparable. Thus, we eliminated the indicators that lacked representativeness and availability.

(3) Choosing the most suitable and representative one

Due to the third criterion, we chose only one indicator for education to make the GDI concise and easily accepted; thus, that indicator had to be the most suitable and representative one. “Literacy rate (adult)” and “Mean years of schooling” are relatively representative and available as education indicators, and they are widely used to measure the education level of a country. We finally chose the “Mean years of schooling” as the education indicator. The first reason is that adult literacy rate is not “fair” for developing countries, and could not represent future education development. Many developing countries became independent after World War II, some even in the 1990s. The older generation in these countries grew up in chaotic wartime, which led to a very low literacy rate. Although the “Mean years of schooling” will be affected by the age structure too, as an average indicator, the impact of age structure on it can be minimized to a large extent. Secondly, adult literacy rate lacks differentiation, especially for countries with a high economic development level, where the level reaches almost 100%. Thirdly, we were able to gather more samples if we chose the “mean years of schooling” indicator.

The selection process of the “education” indicator is briefly described above. It is similar to the selection process of the remaining 9 indicators. Due to the limitation of space, we will not explain the selection process of each indicator in detail. As Table 1 shows, the GDI is a simple and clear systematic composite index with 10 indicators. These indicators are the most basic and primary goals for green development, for the protection of the world's environment, and for sustainable utilization of natural resources.

#### The Entropy Method for Weighting

The entropy method is a more scientific and objective weighting method than other traditional methods (31). There are some popular weighting methods presented in existing literatures, like equal weights, factor analysis, expert weights, and entropy method (8, 9, 32). Among them, equal weights and expert weights methods are lack of objectivity (8). The factor analysis can only estimate weights if correlation exists between indicators (32). The entropy method is a weighting technique that makes weight judgments based on the size of the data information load^1^, which makes it considered as a scientific and objective method compared with other ones (31). According to the principle of entropy method, the weight of each indicator can be calculated through the following steps.

Normalization is the first step. There are many kinds of normalization methods, such as “ranking,” “distance to target,” “Z-score,” “min–max” (34). The min–max method is generally used for normalization in entropy method because it is simple, mature, and widely used (32, 35). The min–max method can also fully reflect the data information load of an indicator, according to the idea and principle of information entropy (33). Before the min–max normalization method, these 12 indicators are divided into “positive indicators” and “negative indicators.” Positive indicators refer to those indicators whose higher values mean better performance of green development, like air quality and education, and negative indicators are the ones whose lower values represent better performance, such as the PM2.5. The min–max normalization formula for positive index and negative index is shown in equation (1) and equation (2) respectively.


(1) $$\begin{array}{l} {\tilde{x}}_{ij}=\frac{X_{ij}-minX_{ij}}{\max X_{ij}-minX_{ij}} \end{array}$$

(2) $$\begin{array}{l} {\tilde{x}}_{ij}=1-\frac{X_{ij}-minX_{ij}}{\max X_{ij}-minX_{ij}} \end{array}$$

In the equations above, *X* is the raw data value, min(*X*) is the minimum observed value of the indicator, max(*X*) is the maximum observed value of the indicator, *X*_*ij*_ is the indicator j of country i, and ${\tilde{x}}_{ij}$ is the result of normalization.

The entropy value *e*_*j*_ of indicator j could be obtained, as shown in equations (3) and (4).


(3) $$\begin{array}{l} k=1/ln\left(n\right) \end{array}$$

(4) $$\begin{array}{l} e_{j}=-k\overset{n}{\underset{i=1}{\sum }}{\tilde{x}}_{ij}\ln{\tilde{x}}_{ij} \end{array}$$

The information utility value of indicator *j* is calculated, namely *g*_*j*_ in equation (5).


(5) $$\begin{array}{l} g_{j}=1-e_{j} \end{array}$$

Finally, we can get the weight of indicator *j*, namely ω_*j*_, as shown in equation (6), and the results of entropy method could be obtained by the Stata 15.0.


(6) $$\begin{array}{l} \omega_{j}=g_{j}/\overset{p}{\underset{j=1}{\sum }}g_{j} \end{array}$$

### Methods for Progress Measure

The progress measure method adopted in our study is based on the method used by Hametner and Kostetckaia (3), that is calculating the compound average growth rate (CAGR) between two points in time. The CAGR technique has 3 main merits: (1) could provide results in %-change per year that can be compared across different time spans (5); (2) its application does not need the existence of quantitative targets (12); (3) it is sufficient if the desired direction in which an indicator should evolve from an green development point of view is known (3). The CAGR formula is shown as equation (7):


(7) $$\begin{array}{l} CAGR={\left(\frac{y_{t}}{y_{t_{0}}}\right)}^{\frac{1}{t-t_{0}}}-\;1, \end{array}$$

where *t*_0_= base year, t= most recent year (that is 2016), *y*_*t*_0__= indicator value in base year, *y*_*t*_ = indicator value in the most recent year (2016).

Referring to the work of Hametner and Kostetckaia (3), we calculate CAGR for two time spans, namely the past 3-year period and the past 6-year period. This distinction provides information about whether a development continues over the whole time period, or whether recent trends have changed (5). Moreover, it can also compare the progress toward green economy in different periods.

### Data and Imputation

This study selects 45 Asian countries from 2010 to 2016 for the green development assessment (see **Table 3** for the list of countries). These countries were selected by two criteria: (1) the data of all 10 indicators is available, for example, North Korea was not selected because of the unavailability of data; (2) internationally recognized non-sovereign entities were not selected, such as Macau, China. In general, the 45 selected countries include most of the Asian countries, covering more than 95% of the population and land in Asia. The data source can be seen in Appendix Table A1.

The current studies prefer to adopt imputation method to fill missing data rather than missing out information (6). So, we adopt various imputation methods to address missing the data missing problem following the actual situation. Firstly, the mean value interpolation method. For example, average value of 2011 and 2013 is used for replacing the value of 2012, if the data of 2011 and 2013 are available, but the data of 2012 is missing. Secondly, the nearest neighbor interpolation method. This method is used to deal with missing data for the variables that are very stable over time, like the arable land. These imputations in instances can distort the results but losing out data might prove costlier to some countries (32).

## Results

### GDI Measurement and Ranking

The weight of each indicator is calculated by entropy method, as shown in the last column of Table 2. As a result, the weights of economic–social and resource–environmental dimensions are 51.58 and 48.42%, respectively. The weights of the two dimensions are very close. It means that stable economic growth, harmonious social development, rational resources utilization, and environmental protection are all important for the green development of a country. From a perspective of indicator, the four indicators with the highest weight are education, energy, health, and income level, whose weights are 14.03, 13.40, 13.16, and 13.02% respectively. It indicates the four indicators are the most important determinants of green development: (1) fair and abundant income is the most basic material security, but also reflects the wealth and capital adequacy of a country; (2) education is an important measure for the accumulation and development of national human capital; (3) health embodies human's basic pursuit for the right to life and the longevity; (4) and energy reflects the demand of current generations for energy consumption, and also reflects the opportunity and guarantee for future generations to utilize energy and develop economy.

**Table 2:** The weight of each indicator in green development index.

**Index**	**Dimension**	**Factor**	**Indicator**	**Weights**
Green development index (GDI)	Economic- social dimension	Economic growth	Real GDP growth	5.34%
		Education	Expected years of schooling	14.03%
		Health	Life expectancy index	13.16%
		Income level	Income index	13.02%
		Economic structure	Employment in services (% of total employment)	6.03%
	Resource- environmental	Climate	CO_2_ emissions per capita	11.01%
	dimension	Air quality	PM2.5	7.56%
		Forest	Forest area (% of total land area)	9.02%
		Arable land	Arable land per person	7.43%
		Energy	Renewable energy consumption (% of total final energy consumption)	13.40%

Table 3 reports the average GDI and its ranking of 45 Asian countries. As a result, the mean value of GDI ranges from 0.3278 to 0.7575. Among these 45 countries, the top five are Singapore (0.7575), Japan (0.7156), Brunei Darussalam (0.6829), Israel (0.6652), and South Korea (0.6536), whereas the bottom five are Syria (0.4483), Pakistan (0.4109), Nepal (0.3676), Yemen (0.3449), and Afghanistan (0.3278).

**Table 3:** The mean value of GDI and its ranking from 2010 to 2016.

**Country**	**GDI**	**Rank**	**Income level**	**Country**	**GDI**	**Rank**	**Income level**
Singapore	0.7575	1	High	Armenia	0.5373	24	Upper-middle
Japan	0.7156	2	High	Qatar	0.5356	25	High
Brunei Darussalam	0.6829	3	High	Iran	0.5327	26	Upper-middle
Israel	0.6652	4	High	Jordan	0.5249	27	Upper-middle
Korea (Rep.)	0.6536	5	High	Azerbaijan	0.5247	28	Upper-middle
Malaysia	0.6454	6	Upper-middle	Kyrgyzstan	0.5100	29	Lower-middle
Turkey	0.6040	7	Upper-middle	India	0.5072	30	Lower-middle
Oman	0.6030	8	High	Vietnam	0.5055	31	Lower-middle
Georgia	0.5874	9	Upper-middle	Bhutan	0.5050	32	Lower-middle
Lebanon	0.5834	10	Upper-middle	Turkmenistan	0.4958	33	Upper-middle
Maldives	0.5753	11	Upper-middle	Mongolia	0.4912	34	Lower-middle
Kuwait	0.5743	12	High	Uzbekistan	0.4781	35	Lower-middle
Indonesia	0.5738	13	Upper-middle	Myanmar	0.4715	36	Lower-middle
United Arab Emirates	0.5665	14	High	Cambodia	0.4652	37	Lower-middle
China	0.5656	15	Upper-middle	Tajikistan	0.4640	38	Low
Saudi Arabia	0.5558	16	High	Bangladesh	0.4627	39	Lower-middle
Lao	0.5502	17	Lower-middle	Iraq	0.4515	40	Upper-middle
Kazakhstan	0.5499	18	Upper-middle	Syria	0.4483	41	Low
Thailand	0.5487	19	Upper-middle	Pakistan	0.4109	42	Lower-middle
Timor-Leste	0.5486	20	Lower-middle	Nepal	0.3676	43	Lower-middle
Sri Lanka	0.5479	21	Lower-middle	Yemen	0.3449	44	Low
Philippines	0.5464	22	Lower-middle	Afghanistan	0.3278	45	Low
Bahrain	0.5435	23	High				

*The income level (2016) is given by the World Bank*.

The GDI ranking of each country showed distinct characteristics in income level. These countries are divided into four categories according to income levels following World Bank's standard (in 2016), namely high, upper-middle, lower-middle, and low income countries. As Table 3 shows, countries with high GDI tended to be with high income level, like the top 5 countries are all high-income countries. On the contrary, most of low-GDI countries are low or lower-middle countries, such as the bottom five ones. This means that there may be a positive correlation between income level and green development level. Of course, income level, as an important subindicator of GDI, is the direct and superficial reasons for this correlation. The fundamental reasons are: (1) those low-income countries have very limited fiscal revenue, leading to insufficient supply of public goods, such as education, medical care, public health, environmental protection, etc. (36). (2) Some developing countries promote economic growth at the cost of resources and environment, while they are inefficient in resource utilization, inadequate in environmental protection and management (37).

However, there are also many exceptions, that is, those rich Middle East countries perform poorly in GDI ranking. For example, Saudi Arabia, Qatar, and Bahrain are global top rich countries with very high per capita GDP and income, but their GDI ranking is, respectively, 16, 23, and 25 among these Asian countries. These countries are considered as high HDI countries because the HDI lacks environmental indicators. But the GDI is a relatively complete green development index, which puts a stop to the “celebration” of “gas-guzzling developed countries” clearly (35, 38).

**Figure 1:**
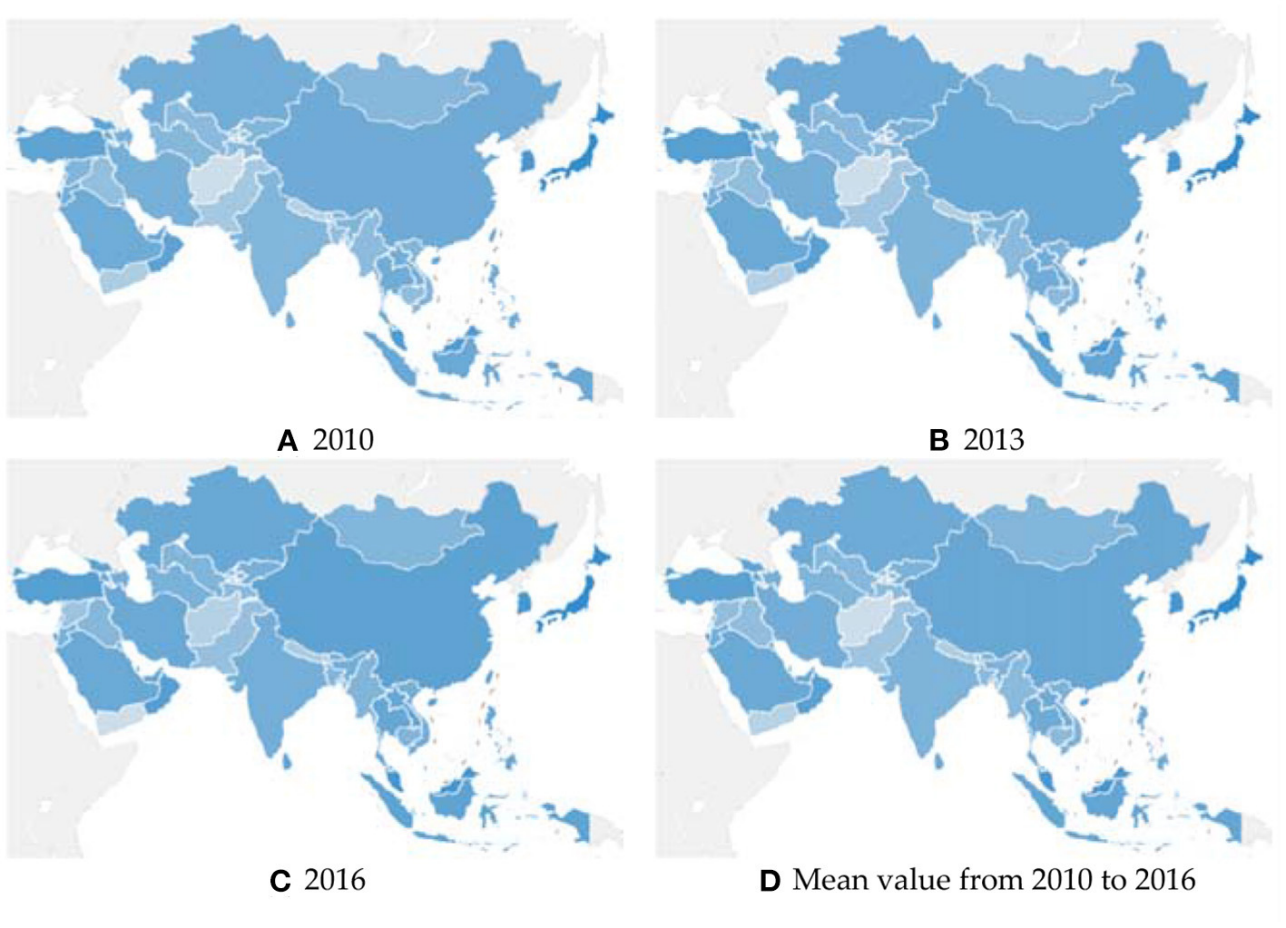
Geographical distribution of GDI in Asia. Subfigure **(A)** is the geographical distribution of GDI in 2010; **(B)** is the geographical distribution of GDI in 2013; **(C)** is the geographical distribution of GDI in 2016; and **(D)** is the geographical distribution of the mean value of GDI from 2010 to 2016.

Figure 1 shows the geographical distribution of GDI in Asia. It should be noted that the darker the blue, the higher the GDI and green development level. Figures 1A–C present the geographical distribution of GDI in 2010, 2013, and 2016, respectively. As a result, GDI ranking always maintains a similar geographical distribution pattern in these three time points. Specifically, the countries in Northeast and Southeast Asia have the deepest blue and the highest green development level, such as Japan, South Korea, and Brunei. The Western Asian countries close to Europe also have good performance in green development, such as Israel. On the contrary, countries in South and Central Asia are the lightest in blue, which means that green development is at the bottom level, such as Nepal and Afghanistan. In sum, the geographical distribution of GDI shows the characteristic, which is high in the east and west Asia, while low in the middle. Moreover, this characteristic is further supported in Figure 1D.

### Progress of GDI in Each Country

**Figure 2:**
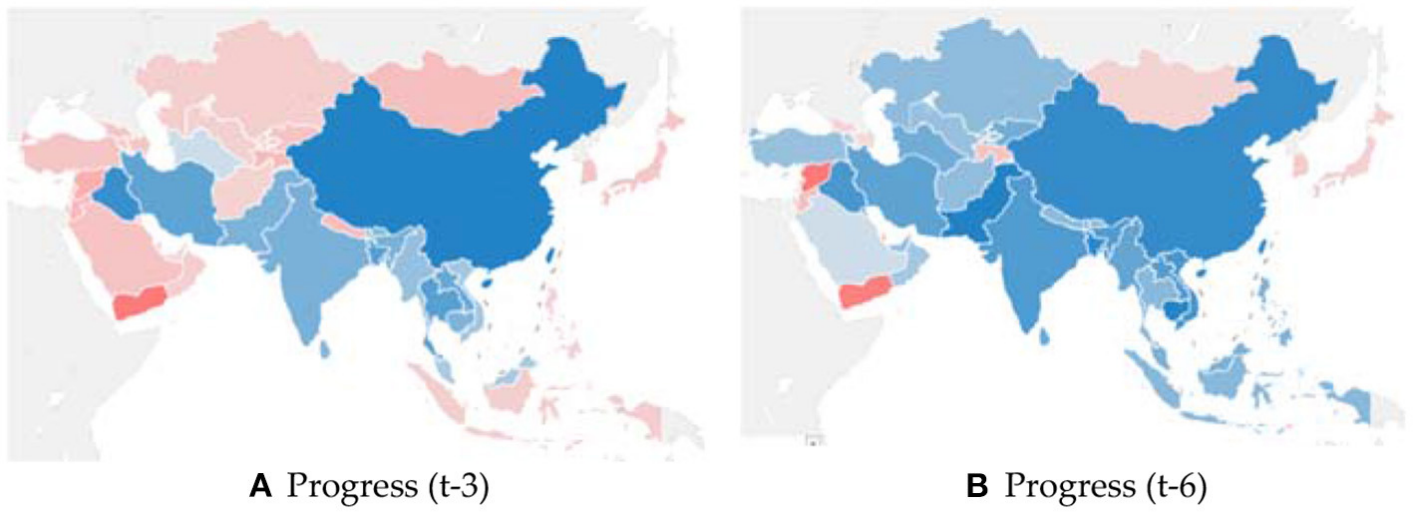
Progress score in different time spans. **(A)** Subfigure a is the geographical distribution of progress score in the past 3-year period (namely 2013–2016), while Subfigure b is that in the past 6-year period (namely 2010–2016), which calculated by the CAGR; **(B)** Blue indicates progress toward to green economy, red indicates movement away from green economy. The darker the color, the stronger the progress or movement away that occurred over the respective time span.

This paper measures the progress toward green economy in Asia countries at different time horizons (t-3 years, t-6 years), as well as the ranking of progress score, based on GDI, *via* the CAGR method. The information of progress score and its ranking are included in Table 4 and Figure 2. “Progress (t-3)” refers to the progress in the past 3-year period (namely 2013–2016), while “Progress (t-6)” refers to that in past 6-year period (namely 2010–2016), which is calculated by the CAGR. Hence, the progress score indicates the progress toward green economy, which is a result in %-change per year. For example, the progress (t-3) score of Japan is −0.78, which means that the CAGR in the past 3-year period is −0.78%. If the progress score is positive, it means that the green development level is improved in the that period, and vice versa. The greater the absolute value of progress score, the faster the improvement or decline of green development level.

**Table 4:** The progress score in different time spans.

**GDI rank (2010–2016)**	**Country**	**Progress (t-3)**		**Progress (t-6)**	
		**Score**	**Rank**	**Score**	**Rank**
1	Singapore	−0.52	25	−0.29	36
2	Japan	−0.78	28	−0.19	34
3	Brunei Darussalam	−1.25	37	−0.37	38
4	Israel	−0.81	30	−0.26	35
5	Korea (Rep.)	−0.78	29	−0.36	37
6	Malaysia	0.12	15	0.46	14
7	Turkey	−0.87	33	0.28	24
8	Oman	−0.39	23	0.19	27
9	Georgia	−0.85	32	−0.10	32
10	Lebanon	−1.69	42	−0.90	41
11	Maldives	−0.57	26	0.12	29
12	Kuwait	−0.32	21	0.45	15
13	Indonesia	−0.38	22	0.38	17
14	United Arab Emirates	−0.11	17	0.26	26
15	China	0.90	1	0.94	4
16	Saudi Arabia	−1.00	35	0.03	30
17	Lao	0.50	6	0.72	8
18	Kazakhstan	−0.48	24	0.33	21
19	Thailand	0.59	4	0.38	18
20	Timor-Leste	−2.50	44	−1.66	43
21	Sri Lanka	0.33	12	0.56	11
22	Philippines	−0.16	18	0.44	16
23	Bahrain	0.40	8	0.33	22
24	Armenia	−0.68	27	0.14	28
25	Qatar	−1.08	36	−1.58	42
26	Iran	0.55	5	0.65	9
27	Jordan	−1.56	41	−0.82	40
28	Azerbaijan	−0.83	31	−0.07	31
29	Kyrgyzstan	−0.92	34	0.52	13
30	India	0.40	7	0.74	7
31	Vietnam	0.16	14	0.80	5
32	Bhutan	0.35	11	0.35	20
33	Turkmenistan	0.01	16	0.53	12
34	Mongolia	−1.29	38	−0.13	33
35	Uzbekistan	−0.30	20	0.28	25
36	Myanmar	0.24	13	0.62	10
37	Cambodia	0.36	10	1.04	2
38	Tajikistan	−1.55	40	−0.66	39
39	Bangladesh	0.61	3	0.99	3
40	Iraq	0.77	2	0.79	6
41	Syria	−2.18	43	−3.52	45
42	Pakistan	0.38	9	1.04	1
43	Nepal	−1.36	39	0.35	19
44	Yemen	−5.35	45	−3.28	44
45	Afghanistan	−0.19	19	0.32	23

*The order of the first column on the right is the ranking of GDI mean in Table 3*.

Table 4 and Figure 2 report the progress scores and rankings of Asian countries in the two time periods. In the past 3-year period, the five countries with the fastest increase in the green development level are China, Iraq, Bangladesh, Thailand, and Iran, while the five countries with the fastest decrease are Jordan, Lebanon, Syria, Timor Leste, and Yemen. In the past 6-year period, the five countries with the fastest progress toward green development are Pakistan, Cambodia, Bangladesh, China, and Vietnam, while the five countries with the fastest decrease are Lebanon, Qatar, Timor Leste, Yemen, and Syria. Among these countries, China and Bangladesh scored high in both periods, while Lebanon, Timor Leste, Yemen, and Syria scored low.

The relationship between GDI ranking and progress score has three interesting characteristics:

First, those GDI leading countries seem to have stagnated at a high level, and some of them even experienced a slight retrogression. For example, Japan, South Korea, and Singapore are all the top-level Asian countries in the green development performance, but their progress scores are negative in the two time periods. The reason why GDI leading countries do not progress overall further toward green economy might be that they have already exploited most of the synergies that exist between the different aspects of green economy, where progress in one area helps achieve progress in another (12). So, those countries have to increasingly face trade-offs between the different aspects of green development, whereby further progress in one aspect is made at the cost of others (39, 40). In the study of European countries, there is a similar phenomenon: sustainable development leading countries, with the highest levels of SDG achievement, have stagnated at this level over the past 15 years, like Sweden, Denmark, and Netherlands (3). It is reasonable to assume that these countries have already reached a high level of green development, so the room for further progress is very limited.

Second, some of countries with medium level GDI have achieved the fastest progress toward green economy. For example, China and Thailand are the countries with the medium level of GDI ranking, but they show a very fast movement toward green economy in both two time periods. This is mainly contributed by the economic growth and environmental protection of these countries in recent years. On the one hand, these countries have achieved rapid economic growth in the past decades, but the insufficient environmental protection and social welfare has led to the medium level of green development. On the other hand, in recent years, these countries have a growing amount of fiscal revenue and enough ability to improve social welfare or govern environment, which makes them have a strong progress momentum toward green economy.

Last, some countries with very low green development level have still moved fast away from green economy. For example, the GDI is rapidly reduced in Syria and Yemen which are definitely low green development countries. “High GDI countries are always alike; but each low GDI country is un-green in its own way”^2^. Some of the countries that are getting worse at green development are due to political instability, such as Syria and Yemen; and some are due to problems in economic system environmental governance. It is clear that some low GDI countries are getting worse in these two periods.

Overall, those green development leading countries seem to have stagnated at this level, and some of them even experienced a slight retrogression; some countries with medium green development level have achieved the fastest progress; whereas the laggards get worse over the past 3- and 6-year periods.

## Discussion: A Comparison Between GDI and Other Indices

The GDI could be seen as an improvement index of the HDI because it is built based on the HDI by adding some indicators with the connotation of green development. Beside the GDI, many researchers have built the improvement indices of HDI by adding environmental indicators, such as the Human Sustainable Development Index (35) and the Human Green Development Index (8). So, we make a comparison about these green development indices.

Since 1990, the HDI is reported annually as part of the Human Development Report of the UNDP, and has gradually become a widely used and cited index for sustainability assessment due to its simple composition and rich connotation. It consists of three (equal weighted) subindices which are aggregated by an arithmetic mean: education, income, and life expectancy. Although the composition is simple, its connotation is very rich. The HDI is based on the theory of welfare economics with fairness and substantial freedom, which contains a deep understanding of the main concept of human development. In the past, the traditional meaning of “development” was strictly economic, as it dealt only with the economic side of development. For instance, per capita GDP used to be a basic indicator for development trend and level. In subsequent years, more and more scholars have moved toward a new concept of development in which economic growth is seen as a condition that is necessary but not sufficient to explain the degree of development of a country. They pay more attention to the real welfare that people enjoy, namely human development. The essential abilities for human development are therefore the abilities to lead a long, healthy life, to obtain knowledge, to access the resources needed for a decent standard of living, and to take part in the life of the community. Based on the above theories and ideas, the HDI is born to measure the human development in national level. Therefore, the HDI gradually becomes one of the most widely used composite index for measuring development.

Human Sustainable Development Index (HSDI), Human Green Development Index (HGDI), and GDI are regarded as improvement indices of the HDI, but they are quite different in composition and connotation. As mentioned earlier, the HDI focuses on the ability and sustainability of human. But no matter the poor, the rich, and even the developing or the developed countries, they must act under the constraints of the earth environment. Human actions and activities are carried out on the earth, and the impact of the actions of each country on its own country is subject to the natural conditions of the world. So, Bravo (35) considers that the environment is also an important part of human sustainable development, and builds the HSDI by adding an indicator (per capita CO_2_ emissions) to present environmental dimension based on the HDI, as is shown in Table 5. Besides, with the process of human development, resource crisis has been exposed, especially the problems of excessive energy consumption and land pollution. Thus, the ability and sustainability of human is under the constraints of the resource on the earth. From these considerations, the HGDI is constructed by adding some indicators both in resource and environmental dimensions (see Table 6). However, green development is to coordinate the economic-social, and resource–environmental development, to balance the intragenerational welfare and maximize the total welfare of generations. Therefore, we should pursue economic growth to ensure the welfare of present generations, while protecting the ecological environment and rationally utilizing the natural resource to ensure the welfare of future generations. If we just want to protect the environment and make the economy stagnate, it is also not a sustainable development mode. Finally, the GDI is built with economic–social and resource–environmental dimensions and 10 indicators (see Table 5).

**Table 5:** The relation and difference of indices.

**Index**	**Indicators**				**Weight**
	**Economic**	**Environmental**	**Social**	**Resource**	
HDI	Income		Education Life expectancy		Equal
HSDI	Income	CO_2_ emissions	Education Life expectancy		Equal
HGDI	Income	CO_2_ emissions PM10 Forest area (%) Proportion of threatened animals (%) Land conservation area (%)	Education Life expectancy Population using improved drinking-water sources (%) Population using improved sanitation facilities (%) Population below the minimum food energy (%)	Utilization ratio of primary energy (%)	Equal
GDI	Income Economic growth Economic structure	CO_2_ emissions PM2.5 Forest area (%)	Education Life expectancy	Renewable energy consumption (%) Arable land	Entropy Method

**Table 6:** The comparison of GDI and other index rankings in 2015.

**Country**	**GDI**	**HDI**	**HSDI**	**HGDI**	**Country**	**GDI**	**HDI**	**HSDI**	**HGDI**
Singapore	1	1	1	6	Timor-Leste	24	33	31	30
Japan	2	2	3	1	Azerbaijan	25	17	10	20
Brunei Darussalam	3	8	22	3	Iran	26	14	9	24
Israel	4	3	2	8	India	27	34	32	40
Korea (Rep.)	5	4	4	2	Jordan	28	24	12	21
Malaysia	6	13	7	4	**Qatar**	29	6	45	41
Turkey	7	15	6	14	Kyrgyzstan	30	31	26	19
Oman	8	10	13	26	Vietnam	31	29	25	12
Georgia	9	16	5	7	Bhutan	32	35	34	5
China	10	20	17	29	Turkmenistan	33	26	30	25
**United Arab Emirates**	11	5	24	23	Mongolia	34	23	18	35
Indonesia	12	28	23	11	Uzbekistan	35	37	36	–
Kuwait	13	11	33	32	Myanmar	36	40	39	27
Maldives	14	25	19	16	Cambodia	37	39	38	22
Lebanon	15	18	11	17	Bangladesh	38	38	37	37
Lao	16	36	35	9	Tajikistan	39	32	29	28
Kazakhstan	17	12	16	18	Iraq	40	30	28	39
Thailand	18	22	14	10	Pakistan	41	42	41	38
Philippines	19	27	21	15	Syria	42	44	44	–
Sri Lanka	20	21	20	–	Nepal	43	41	40	34
Bahrain	21	9	27	31	Afghanistan	44	43	42	43
**Saudi Arabia**	22	7	15	33	Yemen	45	45	43	42
Armenia	23	19	8	13					

*The data of HGDI is not available for Uzbekistan, Syria and Sri Lanka*.

The ranking results of above indices are shown in Table 6. We find that the ranking results of these three green development indices are quite different with that of HDI, especially for the Middle east countries. It shows that the Middle east countries have a high HDI ranking and low GDI, HSDI, and HGDI ranking. For example, Saudi Arabia ranks 6 in HDI, while GDI, HSDI, and HGDI rank 29, 45, and 41, respectively (see Table 6). This is mainly because the HDI does not include environmental indicators, whereas HSDI, HGDI, and GDI do. It is thus clear that the GDI, HSDI, HGDI put a stop to the “celebration” of “gas-guzzling developed countries”.

From the analysis above, GDI, HSDI, and HGDI are all modifications or improvements of HDI. The HSDI adds per capita CO_2_ emissions to HDI, which is a breakthrough of HDI in the environmental dimension. The HGDI has a number of resource and environmental indicators, which can not only reflect sustainable development in the environmental dimension, but also represent the sustainable utilization of resources, while the GDI fully considers the dimensions of economy, society, and resources and environment. Moreover, the GDI adopts the entropy method to weight all subindicators, which represents a scientific and objective method compared with equal weighted method. To sum up, the GDI represents a small step ahead from the HDI, HSDI, and HGDI.

## Conclusion

The purpose of our study is to assess Asian countries' green development performance, and also the progress toward green economy over time. Although the result shows that the northeast Asian countries together with the Singapore and Israel are leaders in green development performance across the Asia, we find that the most progress toward green economy over the past 3- and 6-year periods has been achieved by some medium green development level countries, like China and Bangladesh, while some laggard countries get worse in green development, such as Syria and Yemen. It indicates that the leading countries have reached a high level of green development, and the medium ones move fast toward green economy, while the laggards get worse over the past 3- and 6-year periods.

This paper further demonstrates the viewpoint of Hametner and Kostetckaia (3), that is, it is not sufficient to calculate composite development indices and rankings at a time point or period, and necessary to monitor progress toward green economy over time. As our result shows, a country is a green development leader or laggard does not mean that it can be guaranteed to achieve the fastest progress. In other words, it's uncertain that whether leading green development countries can maintain the progress toward green economy, or whether the laggards have the higher potential for progress. Therefore, monitor progress is necessary.

We derived some policy implications for public health based on our research:

First, clarifying the concept of green economy and strengthening the idea of green economy will helps to cultivate public awareness of environmental protection and environmental ethics, leading to a good public health state. The cultivation of public awareness of environmental protection needs public opinion to make green development a broad consensus. Therefore, to make clear what is green economy or development is the prerequisite for public to understand and put it into practice.

Second, strengthening the idea of green economy is helpful to cultivate the public green consumption that increase the quality level of public health (41). Green consumption is considered to be a consumption mode conducive to ecological and environmental protection, such as driving electric vehicles instead of gasoline vehicles. With the rapid economic growth, the consumption level of consumers has been greatly improved. The public demand for electronic products, plastic products, rubber products and disposable products is increasing (42). This will inevitably exert pressure on natural resources and ecological environment (43). Therefore, to strengthen and publicize the concept of green economy is helpful to cultivate public green consumption psychology, such as reducing the consumption of plastic products (41).

Last, green consumption will further improve the green production willingness of enterprises. Green production refers to an environment-friendly production process or output with high efficiency and low pollution (44). The purpose of enterprise is to meet the needs of consumers and then maximize profits. When green economy and green consumption become the consensus of most people, enterprises will improve production technology, reform production mode, and provide green products to meet green consumption demand, so as to reduce environmental pollution (45). It is beneficial for public health soundness. For example, the market of green food and organic food is becoming larger and larger, which may be beneficial to green development.

## Data Availability Statement

Publicly available datasets were analyzed in this study. This data can be found here: https://www.sustainabledevelopmentindex.org.

## Author Contributions

MS conceived, designed the research, provided guidance throughout the entire research process, and wrote and supplemented the English paper. MJ participated in data analysis. HJ and F-ST reviewed and edited and paper and are responsible for all R&R works. All authors contributed to the article and approved the submitted version.

## Funding

The authors acknowledge funding support from the Major Program Project of the National Social Science Fund of China (No: 19ZDA055), Zhejiang Sci-Tech University (ZSTU) Scientific Research Fund (No: 21092117-Y), and ZSTU Philosophy and Social Sciences Research Prosperity Program (No: 21096075-Y).

## Conflict of Interest

The authors declare that the research was conducted in the absence of any commercial or financial relationships that could be construed as a potential conflict of interest. The reviewer SG declared a past co-authorship with one of the authors MJ to the handling editor.

## Publisher's Note

All claims expressed in this article are solely those of the authors and do not necessarily represent those of their affiliated organizations, or those of the publisher, the editors and the reviewers. Any product that may be evaluated in this article, or claim that may be made by its manufacturer, is not guaranteed or endorsed by the publisher.

## Footnotes

^1^Entropy Method is a weighting technique based on the idea of entropy from information theory. Specifically, information is a measure of the order degree and entropy is a measure of the disorder degree in a system; hence, the smaller entropy of the indicator, the more information provided by the indicator, the greater its role and weight in the comprehensive evaluation (31, 33). As Zhang et al. (33) have pointed, the weight measured by the Entropy Method represents the relative rate of change of the indicator in a composite index system, while the relative level of each indicator should be figured by the standardized value of its data.

^2^Similar to Leo Tolstoy's famous remark “Happy families are all alike; each unhappy family is unhappy in its own way”.
