# Supplementary material for: How Fast Are the Asian Countries Progressing Toward Green Economy? Implications for Public Health
Source: Front Public Health. 2022 Feb 7;9:753338. doi: 10.3389/fpubh.2021.753338 (PMC8858809; doi:10.3389/fpubh.2021.753338)
Supplement: Supplementary file 1 [file Table_1.docx]

Appendix

**Table A1.** The descriptions and data sources of the 10 indicators in GDI

| **Indicator** | **Description and Source** |
| --- | --- |
| GDP Growth | Real GDP growth.  *Data source: World Bank.* |
| Income index | According to Atkinson (1970), calculating the income index can reflect fairness and equality, in the case of unequal distribution factors, based on the disposable income or consumption of per capita family. The higher the income index is, the better the economic situation of the country is, and the more equal and fairer the income distribution of country is.  *Data source: UNDP.* |
| Employment in services (% of total employment) | The proportion of employments of the tertiary industry in total employments which is used to measure the economic structure.  *Data source: UNDP.* |
| Per capita CO_2_ emissions | It refers to the CO2 emission generated by the combustion of energy such as coal, oil, natural gas and so on (Unit: ton per person).  *Data source: IEA.* |
| PM2.5 | It represents the concentration of fine suspended particles with a diameter less than 2.5 microns in the atmosphere, which can penetrate the respiratory tract and cause serious health damage (Unit: microgram / m3).  *Data source: World Bank.* |
| Forest coverage rate | The forest coverage rate is the proportion of forest area in the total land area. While the forest area refers to the land covered by upright trees (at least 5m) which grow naturally or are planted artificially.  *Data source: UNDP.* |
| Arable land per person | Arable land includes temporary crop land (double cropping rice field is calculated once), temporary grassland for mowing or pasture, market or kitchen garden land and temporary fallow land, but excludes the land abandoned due to rotation.  *Data source: World Bank.* |
| Renewable energy consumption | It refers to the proportion of renewable energy consumption in total energy consumption. The higher the proportion is, the more conducive to the sustainable development in resources and environmental dimension.  *Data source: UNDP.* |
| Expected years of schooling | Expected year of education (unit: years). Number of years of schooling that a child of school entrance age can expect to receive if prevailing patterns of age-specific enrolment rates persist throughout the child’s life.  *Data source: UNDP.* |
| Life expectancy index | According to Atkinson (1970), calculating the life expectancy index can reflect fairness and equality, in the case of unequal distribution factors, based on the data of UN life table. The higher the index value, the better the health status of residents, the more equal and fairer the access to health for residents.  *Data source: UNDP.* |

Note: the UNDP and IEA are short for the United Nations Development Program and the International Energy Agency, respectively.

**Table A2.** The measurement of GDI, 2010-2016.

| **R** | **Country** | **2010** | **2011** | **2012** | **2013** | **2014** | **2015** | **2016** |
| --- | --- | --- | --- | --- | --- | --- | --- | --- |
| 1 | Singapore | 0.7646 | 0.7722 | 0.7404 | 0.7633 | 0.7704 | 0.7401 | 0.7514 |
| 2 | Japan | 0.7145 | 0.7329 | 0.7028 | 0.7231 | 0.7248 | 0.7051 | 0.7064 |
| 3 | Brunei Darussalam | 0.6824 | 0.7015 | 0.6747 | 0.6928 | 0.6882 | 0.6737 | 0.6672 |
| 4 | Israel | 0.6656 | 0.6884 | 0.6475 | 0.6715 | 0.6773 | 0.6505 | 0.6554 |
| 5 | Korea (Rep.) | 0.6571 | 0.6727 | 0.6416 | 0.6583 | 0.6644 | 0.6379 | 0.643 |
| 6 | Malaysia | 0.6345 | 0.6524 | 0.6227 | 0.6498 | 0.6609 | 0.6453 | 0.6522 |
| 7 | Turkey | 0.5925 | 0.6155 | 0.5777 | 0.6186 | 0.6212 | 0.6010 | 0.6026 |
| 8 | Oman | 0.5960 | 0.6139 | 0.5877 | 0.6098 | 0.6136 | 0.5977 | 0.6027 |
| 9 | Georgia | 0.5864 | 0.5966 | 0.5644 | 0.5981 | 0.6033 | 0.5798 | 0.5830 |
| 10 | Lebanon | 0.5961 | 0.6096 | 0.5749 | 0.5940 | 0.5861 | 0.5590 | 0.5644 |
| 11 | Maldives | 0.5698 | 0.5918 | 0.5556 | 0.5839 | 0.5883 | 0.5635 | 0.5739 |
| 12 | Kuwait | 0.5575 | 0.5967 | 0.5572 | 0.5782 | 0.5932 | 0.5648 | 0.5726 |
| 13 | Indonesia | 0.564 | 0.5857 | 0.5559 | 0.5837 | 0.5847 | 0.5654 | 0.5771 |
| 14 | United Arab Emirates | 0.5566 | 0.585 | 0.5465 | 0.5673 | 0.5785 | 0.5664 | 0.5655 |
| 15 | China | 0.5517 | 0.5651 | 0.5392 | 0.5679 | 0.5843 | 0.5678 | 0.5834 |
| 16 | Saudi Arabia | 0.5462 | 0.5757 | 0.539 | 0.5638 | 0.5822 | 0.5369 | 0.5471 |
| 17 | Lao | 0.5378 | 0.5564 | 0.5244 | 0.5532 | 0.5653 | 0.5525 | 0.5616 |
| 18 | Kazakhstan | 0.5378 | 0.5591 | 0.5328 | 0.5566 | 0.5676 | 0.5465 | 0.5487 |
| 19 | Thailand | 0.5456 | 0.5579 | 0.5274 | 0.5483 | 0.5576 | 0.5463 | 0.5580 |
| 20 | Timor-Leste | 0.5686 | 0.5896 | 0.5447 | 0.5550 | 0.5478 | 0.5197 | 0.5144 |
| 21 | Sri Lanka | 0.5385 | 0.5596 | 0.527 | 0.5514 | 0.5602 | 0.5414 | 0.5568 |
| 22 | Philippines | 0.5377 | 0.554 | 0.5253 | 0.5545 | 0.5594 | 0.5423 | 0.5519 |
| 23 | Bahrain | 0.5381 | 0.5569 | 0.5235 | 0.5423 | 0.5551 | 0.5401 | 0.5488 |
| 24 | Armenia | 0.5284 | 0.5508 | 0.5182 | 0.5441 | 0.5505 | 0.5362 | 0.5330 |
| 25 | Qatar | 0.5677 | 0.5668 | 0.5241 | 0.5330 | 0.5383 | 0.5036 | 0.5159 |
| 26 | Iran | 0.5249 | 0.5456 | 0.5176 | 0.5368 | 0.5456 | 0.5129 | 0.5457 |
| 27 | Jordan | 0.5325 | 0.5506 | 0.5169 | 0.5311 | 0.5299 | 0.5067 | 0.5067 |
| 28 | Azerbaijan | 0.5223 | 0.5341 | 0.5064 | 0.5333 | 0.5367 | 0.5196 | 0.5202 |
| 29 | Kyrgyzstan | 0.4944 | 0.5228 | 0.4903 | 0.5243 | 0.5254 | 0.5028 | 0.5099 |
| 30 | India | 0.4943 | 0.5116 | 0.487 | 0.5106 | 0.5230 | 0.5074 | 0.5168 |
| 31 | Vietnam | 0.4901 | 0.5153 | 0.4863 | 0.5116 | 0.5187 | 0.5027 | 0.5141 |
| 32 | Bhutan | 0.5027 | 0.5126 | 0.4818 | 0.5078 | 0.5165 | 0.5001 | 0.5132 |
| 33 | Turkmenistan | 0.4856 | 0.5051 | 0.4725 | 0.5012 | 0.5132 | 0.4916 | 0.5013 |
| 34 | Mongolia | 0.4839 | 0.5097 | 0.4677 | 0.4993 | 0.5139 | 0.4841 | 0.4802 |
| 35 | Uzbekistan | 0.4727 | 0.4867 | 0.4572 | 0.485 | 0.4917 | 0.4725 | 0.4806 |
| 36 | Myanmar | 0.4636 | 0.4772 | 0.4461 | 0.4776 | 0.4847 | 0.4703 | 0.4812 |
| 37 | Cambodia | 0.4502 | 0.4713 | 0.4407 | 0.4738 | 0.4786 | 0.4626 | 0.4791 |
| 38 | Tajikistan | 0.4741 | 0.4812 | 0.4461 | 0.4774 | 0.4681 | 0.4459 | 0.4555 |
| 39 | Bangladesh | 0.4512 | 0.4704 | 0.4362 | 0.4702 | 0.4722 | 0.4601 | 0.4788 |
| 40 | Iraq | 0.4455 | 0.4662 | 0.433 | 0.4564 | 0.456 | 0.4366 | 0.4671 |
| 41 | Syria | 0.4953 | 0.5263 | 0.4768 | 0.4267 | 0.4099 | 0.4035 | 0.3994 |
| 42 | Pakistan | 0.3963 | 0.4187 | 0.3902 | 0.417 | 0.4201 | 0.4119 | 0.4218 |
| 43 | Nepal | 0.3552 | 0.3795 | 0.3501 | 0.3779 | 0.3834 | 0.3648 | 0.3627 |
| 44 | Yemen | 0.3775 | 0.3697 | 0.341 | 0.3644 | 0.3548 | 0.2979 | 0.309 |
| 45 | Afghanistan | 0.3288 | 0.3229 | 0.3056 | 0.3371 | 0.3425 | 0.3225 | 0.3352 |
